# Use of Four Next-Generation Sequencing Platforms to Determine HIV-1 Coreceptor Tropism

**DOI:** 10.1371/journal.pone.0049602

**Published:** 2012-11-14

**Authors:** John Archer, Jan Weber, Kenneth Henry, Dane Winner, Richard Gibson, Lawrence Lee, Ellen Paxinos, Eric J. Arts, David L. Robertson, Larry Mimms, Miguel E. Quiñones-Mateu

**Affiliations:** 1 Computational and Evolutionary Biology, Faculty of Life Sciences, The University of Manchester, Manchester, United Kingdom; 2 Institute of Organic Chemistry and Biochemistry, Prague, Czech Republic; 3 Division of Infectious Diseases, Case Western Reserve University, Cleveland, Ohio, United States of America; 4 Department of Pathology, Case Western Reserve University, Cleveland, Ohio, United States of America; 5 Pacific Biosciences, Menlo Park, California, United States of America; 6 Quidel Corporation, San Diego, California, United States of America; 7 University Hospital Translational Laboratory, University Hospitals Case Medical Center, Cleveland, Ohio, United States of America; INSERM, France

## Abstract

HIV-1 coreceptor tropism assays are required to rule out the presence of CXCR4-tropic (non-R5) viruses prior treatment with CCR5 antagonists. Phenotypic (e.g., Trofile™, Monogram Biosciences) and genotypic (e.g., population sequencing linked to bioinformatic algorithms) assays are the most widely used. Although several next-generation sequencing (NGS) platforms are available, to date all published deep sequencing HIV-1 tropism studies have used the 454™ Life Sciences/Roche platform. In this study, HIV-1 co-receptor usage was predicted for twelve patients scheduled to start a maraviroc-based antiretroviral regimen. The V3 region of the HIV-1 *env* gene was sequenced using four NGS platforms: 454™, PacBio® *RS* (Pacific Biosciences), Illumina®, and Ion Torrent™ (Life Technologies). Cross-platform variation was evaluated, including number of reads, read length and error rates. HIV-1 tropism was inferred using Geno2Pheno, Web PSSM, and the 11/24/25 rule and compared with Trofile™ and virologic response to antiretroviral therapy. Error rates related to insertions/deletions (indels) and nucleotide substitutions introduced by the four NGS platforms were low compared to the actual HIV-1 sequence variation. Each platform detected all major virus variants within the HIV-1 population with similar frequencies. Identification of non-R5 viruses was comparable among the four platforms, with minor differences attributable to the algorithms used to infer HIV-1 tropism. All NGS platforms showed similar concordance with virologic response to the maraviroc-based regimen (75% to 80% range depending on the algorithm used), compared to Trofile (80%) and population sequencing (70%). In conclusion, all four NGS platforms were able to detect minority non-R5 variants at comparable levels suggesting that any NGS-based method can be used to predict HIV-1 coreceptor usage.

## Introduction

The discovery that human immunodeficiency virus type 1 (HIV-1) requires a co-receptor to enter target cells, mainly the chemokine receptors CCR5 or CXCR4 [1], [2], was not only crucial to better understand HIV-1 transmission and pathogenesis but opened the door for the designing of novel antiretroviral drugs targeting host cell entry. Multiple strategies to block the replication of CCR5- or CXCR4-tropic (R5 or X4, respectively) viruses have been studied [3], leading to the approval for clinical use of the first CCR5-receptor antagonist (maraviroc, Selzentry/Celsentri, Pfizer, NY) in 2007 [4]. Like other co-receptor antagonists in development, maraviroc's activity is very specific, showing no direct activity against viruses able to use CXCR4 to enter the target cell [5], [6]. Thus, an HIV-1 tropism test should be performed prior to initiation of maraviroc-containing regimens to rule out the presence of detectable non-CCR5-tropic (non-R5) virus [7], [8], [9].

Several phenotypic and genotypic assays have been developed to assess HIV-1 co-receptor usage or tropism [7]. Most phenotypic assays involve the generation of patient-derived *env*-recombinant viruses to determine their ability to infect reporter cell lines expressing HIV-1 receptors and co-receptors [10], [11], [12], [13]. The new version of Trofile™ (Monogram Biosciences, South San Francisco, CA) [11], i.e., the Enhanced Sensitivity Trofile Assay (ESTA) [14] is currently the most widely used HIV-1 coreceptor tropism assay. Nonetheless, phenotypic assays share a few practical limitations such as high cost and long turnaround time, which restrict their use and consequentially hinder access to future CCR5 antagonists/agonists.

Genotypic tests are a faster, less expensive alternative to inferring HIV-1 coreceptor tropism from *env* sequences [7], [8]. Considerable effort has been made to develop genotypic assays able to predict HIV-1 co-receptor usage based on just the V3 region of the *env* gene [7], [9], [15], which seems to be the principal determinant of HIV-1 tropism [16], [17]. However, genotypic tests based on bulk capillary electrophoresis (Sanger) sequencing of a population of V3 sequences lack the sensitivity to detect minority variants present below 20% of the viral population [18], [19], [20], [21]. For that reason, several studies have evaluated the use of next-generation (NGS) or deep sequencing to detect minority non-R5 HIV-1 variants [22], [23], [24], [25], [26], [27], [28] or low frequency drug-resistant variants that could lead to treatment failure [29], [30], [31], [32], [33]. Prediction of HIV-1 coreceptor usage by deep sequencing is highly concordant with phenotypic assays (82% to 87%) [25], [28], [34], has improved sensitivity for detecting non-R5 variants over population sequencing [22], [25], [27], [35], [36], and predicts the success of maraviroc-based antiretroviral regimens [25], [28].

To date all published HIV-1 deep sequencing studies have used the 454™ Life Sciences platform (454 Life Sciences/Roche, Branford, CT); some of which were focused on HIV-1 tropism prediction [22], [23], [24], [25], [27], [28], [34], [35], [37], [38]. The advent of novel NGS technologies offering different chemistries, simplified sample preparation, faster turnaround times, and reduced cost per bp sequenced prompted us to compare the ability of four NGS platforms, i.e., 454™ Life Sciences/Roche, Illumina® (Illumina, Inc. San Diego, CA), PacBio® *RS* (Pacific Biosciences, Menlo Park, CA), and Ion Torrent™ (Ion Torrent/Life Technologies, South San Francisco, CA) to determine HIV-1 coreceptor tropism.

## Materials and Methods

### Clinical samples

Twelve RNA specimens, derived from plasma samples collected from HIV-infected individuals prior to enrollment in the (i) maraviroc expanded access program (EAP) at multiple centers in Europe or (ii) ALLEGRO trial, a multicenter study to assess the prevalence of R5 HIV-1 variants in Spain [39], were obtained from the Hospital Carlos III (Madrid, Spain) [21]. Phenotypic HIV-1 coreceptor tropism was determined at baseline using the original version of the Trofile™ assay (Monogram Biosciences), which had a reported non-R5 variant detection limit of 5 to 10% [11]. Written informed consent was obtained from the patients before participation in the study as previously described [21], [39].

### RT-PCR amplification

**Figure 1:**
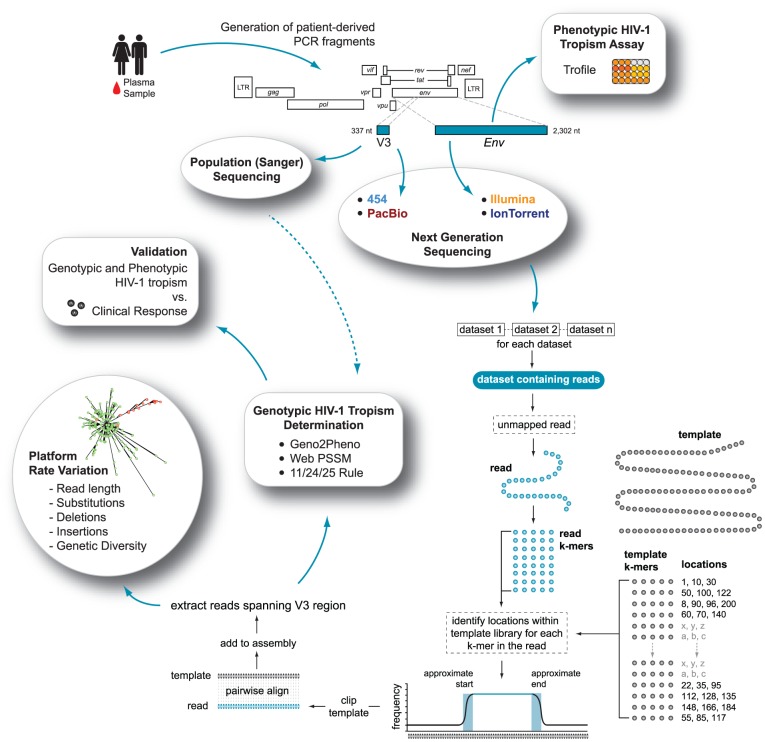
Schema summarizing the strategy followed in this study to compare the use of the four next-generation sequencing platforms (454™, Illumina®, PacBio®, and Ion Torrent™) to determine HIV-1 coreceptor tropism (see text for full details).

Viral RNA was reverse-transcribed using AccuScript High Fidelity Reverse Transcriptase (Stratagene Agilent; Santa Clara, CA) and the corresponding antisense external primer in 20-µl reaction mixture containing 1 mM dNTPs, 10 mM DTT and 10 units of RNase inhibitor. Viral cDNA was then PCR amplified using a series of external and nested primers with defined cycling conditions. Using the same external (first-round) PCR reactions that covered the entire HIV-1 envelope (*env*) gene (2,830 nt), two different PCR fragments were amplified due to intrinsic requirements of each NGS platform (Fig. 1). These twelve samples are part of a larger cohort analyzed in a separate study (Weber and Quinones-Mateu, submitted for publication). In that study 105 patient-derived 337 bp amplicons corresponding to a short region around the HIV-1 V3 loop were analyzed with the 454™ system. Here, we were able to sequence the same amplicons using the PacBio® *RS* platform; however, the Illumina® and Ion Torrent™ systems used in this study are better suited to sequence longer DNA regions, shearing them into small fragments (150 to 200 bp). Processing and sequencing the small 337 bp PCR products may have been difficult with these two platforms. Thus, 337-nucleotide (nt) fragments encompassing the V3 region were generated in single nested (second-round) PCR reactions. These small amplicons were sequenced using 454™ and PacBio® *RS* sequencing systems. A larger fragment corresponding to the *env* gene was amplified as a 2,302 nt fragment, that is, all the surface glycoprotein (gp120) and most of the transmembrane glycoprotein (gp41), missing only 321 nt of the gp41 cytoplasmic domain. These amplicons were sequenced using Illumina® and Ion Torrent™ platforms. External PCR reactions were carried out in a 50-µl mixture containing 0.2 mM dNTPs, 3 mM MgCl_2_ and 2.5 units of Pfu Turbo DNA Polymerase (Stratagene). Nested PCR reactions for population sequencing analysis were carried out in 50-µl mixture containing 0.2 mM dNTPs, 0.3 units of Pfu Turbo DNA Polymerase and 1.9 units of Taq Polymerase (Denville Scientific; Metuchen, NJ), then purified with the QIAquick PCR Purification kit (Qiagen) and quantified with Quant-iT PicoGreen dsDNA kit (Invitrogen). Nested PCR reactions for deep sequencing analysis were customized for each NGS system as described below.

### Population (standard Sanger) sequence analysis

PCR products corresponding to the gp120/gp41-coding regions of HIV-1 were purified with the QIAquick PCR Purification kit (Qiagen) and the V3 region sequenced (population or global sequence) using AP Biotech DYEnamic ET Terminator cycle with Thermosequenase II (Davis Sequencing LCC, Davis, CA) (Fig. 1). Nucleotide sequences were analyzed using DNASTAR Lasergene Software Suite v.7.1.0 (Madison, WI).

### Deep sequencing of the V3 region of *env*

Second-round PCR amplification and deep sequencing analysis was customized for each NGS platform as follows:

*454/Roche*: A 337-nt fragment encompassing the V3 region was generated by nested PCR from an external PCR product containing the entire *env* gene (2,830 nt) as described above. Single nested PCR reactions were carried out with Phusion High-Fidelity DNA Polymerase (New England Biolabs, Ipswich, MA) in a 50 µl-reaction mixture containing 0.2 mM dNTPs, 2.5 mM MgCl_2_, and 0.2 µM of both the antisense and barcoded sense primers. PCR products were purified with the QIAquick PCR Purification kit (Qiagen) and quantified with Quant-iT PicoGreen dsDNA kit (Invitrogen). Pooled PCR products were clonally amplified on capture beads in water-oil-emulsion micro-reactors. A total of 500,000 HIV-1 *env* enriched-DNA beads were deposited in the wells of a 454 GS FLX instrument (454 Life Sciences/Roche) and pyrosequenced in forward direction using 200 cycles in a ten-hour sequencing run.
*Illumina*: A 2,302 nt fragment of the *env* gene was amplified from an external PCR product as described above for the population sequencing, then purified (QIAquick PCR Purification, Qiagen) and quantified (Quant-iT PicoGreen dsDNA, Invitrogen). Paired-end DNA libraries were prepared using Nextera DNA sample prep kit (Epicentre Biotechnologies, Madison, WI) according to manufacturer's protocol. Briefly, 50 ng of PCR product were subjected to tagmentation with high-molecular-weight buffer and Nextera enzyme mix at 55°C for 5 minutes followed by limited nine-cycle PCR using half of the purified tagmentation reaction with Nextera PCR mix. Amplicons were then purified using Zymo Clean-up kit (Zymo Research, Irvine, CA), shipped to Illumina, Inc. (San Diego, CA) and used as input for bPCR and cluster preparation according to standard Illumina protocol. Amplicons were sequenced on the Illumina Genome Analyzer IIx (GAIIx) using version 5 chemistry.
*Pacific Biosciences*: A 337 nt fragment encompassing the V3 region was generated by nested PCR from a external PCR product containing the entire *env* gene (2,830 nt) as described above for 454/Roche. Unpurified PCR products were shipped to Pacific Biosciences (Menlo Park, CA) where, after visualization by electrophoresis through 1.2% agarose gel (Lonza Flashgel), the PCR reactions were purified (QIAquick PCR Purification, Qiagen) according to the manufacturer's instructions. Each sample was eluted into 30 µl of EB buffer (10 mM Tris pH 8.0), re-visualized by electrophoresis, then quantified using UV spectrophotometry (NanoDrop, Thermo Scientific, Wilmington, DE). A total of 2 µg for each sample was used as input into library preparation using standard Pacific Biosciences version C1 chemistry, with the following modifications: templates were annealed at 40 nM DNA concentration, 80 nM primer concentration, and Polymerase:Template binding reactions were at 18 nM using ECR2 enzyme (Pacific Biosciences). The samples were sequenced on the PacBio RS running version 1.2.1 software with the standard 2×45 minute collection protocol, and one SMRTCell was used for each sample. Circular consensus sequencing (CCS) reads were generated from zero mode waveguides (ZMW) containing three or more sequencing passes across the template.

#### Ion Torrent

A 2,302 nt fragment of the *env* gene was amplified from an external PCR product, then purified (QIAquick PCR Purification, Qiagen) and quantified (Quant-iT PicoGreen dsDNA, Invitrogen) as described above for Illumina. The Ion Xpress™ Fragment Library Kit (Life Technologies, Carlsbad CA) was used to construct a library for shotgun sequencing on the Ion Personal Genome Machine (PGM, Ion Torrent/Life Technologies). Briefly, amplicon DNA was randomly fragmented using the Ion Shear™ Plus Reagent (Life Technologies). The P1 adapter (5′-CCA CTA CGC CTC CGC TTT CCT CTC TAT GGG CAG TCG GTG AT; 5′-ATC ACC GAC TGC CCA TAG AGA GGA AAG CGG AGG CGT AGT GG*T*T) or one of 12 A_BC adapters were then ligated to the repaired fragment ends. Following ligation and size selection (i.e., 150+/−20 nucleotides; Pippin Prep™, Life Technologies) the library was PCR amplified using forward (5′-CCA TCT CAT CCC TGC GTG TC) and reverse (5′-CCA CTA CGC CTC CGC TTT CCT CTC TAT G) primers. The quality and quantity of each of the 12 libraries was assessed with the 2100 Bioanalyzer (DNA High Sensitivity Chip, Agilent Technologies, Sunnyvale CA). Templates were then prepared and enriched for sequencing on the Ion Sphere Particles™ (ISPs) using the Ion Xpress™ Template Kit (Life Technologies) prior to sequencing on the Ion PGM with the Ion Sequencing Kit (Life Technologies). Fifteen million templated ISPs were primed with the Ion Sequencing primer (5′-CCA TCT CAT CCC TGC GTG TCT CCG AC) and then mixed with the Ion Sequencing Polymerase. The primer-activated polymerase-bound ISPs were loaded into the Ion 314™ Chip (Life Technologies) and subjected to 65 cycles of sequencing with the standard nucleotide flow order. Signal processing and base calling was performed with Torrent Analysis Suite version 1.5.

Finally, it is important to note that the 454™ sequencing was performed in the laboratory of Dr. Hendrik Poinar (McMaster University, Hamilton, Canada), while for Illumina®, PacBio®, and Ion Torrent™ the PCR products were sent for sequencing at the respective company. V3 nucleotide sequences obtained by deep sequencing using any of the NGS platforms (as described below) have been submitted to the Los Alamos National Laboratory HIV-DB Next Generation Sequence Archive (http://www.hiv.lanl.gov/content/sequence/HIV/NextGenArchive/Archer2012).

### Read mapping, variant sensitivity and phylogenetic inference

To minimize the amount of data loss due to high sequence variability and to allow for interpatient indel variation across the V3 region, sample-specific reference sequences were constructed as previously described [24]. First, sequences corresponding to the HIV genomic region spanning positions 6,900 to 7,400 on the HXB2 reference strain (accession no: K03455) were extracted, followed by replacement with the V3 population sequence derived from each sample. For each sample, reads derived from the four NGS platforms were then independently mapped to the respective sample-specific reference sequence (Fig. 1) and all indel and substitution information in relation to the reference sequenced stored as described [40]. For each dataset, reads spanning the V3 region (coordinates 210 to 315 within the reference templates) were extracted, truncated and translated for genotyping. Within each dataset only one representative of any identical variant was maintained, but the overall frequency stored. Finally, all variants with a frequency >5 within the population, calculated by each platform, were combined and clustered using a neighbor-joining algorithm implemented within Segminator II [40].

### HIV-1 coreceptor tropism determination

HIV-1 co-receptor tropism was predicted from population and extracted V3 read data as described above using several bioinformatics tools (Fig. 1). In the case of global sequences, nucleotide mixtures were considered when the second highest peak in the electropherogram was above 25%, and then these nucleotide mixtures were translated into all possible permutations. The algorithms used to infer HIV-1 tropism from V3 amino acid sequences were: (i) Geno2Pheno [41], with false positive rates (FPR, i.e., predicted frequency of classifying an R5 sequence as non-R5 virus) based on optimized cutoffs for determining HIV-1 coreceptor usage (3.5%) as previously described [25], [28], [42] or the recommendation from the European Consensus Group on clinical management of HIV-1 tropism testing (10%) as described in the Geno2Pheno website (http://coreceptor.bioinf.mpi-inf.mpg.de/index.php), (ii) Web PSSM using the subtype B x4r5 matrix [43], and (iii) the 11/24/25 charge rule [44], [45] implemented within our analysis pipeline. Finally, plasma samples were classified as containing non-R5 viruses if at least 2% of the individual sequences, as determined by deep sequencing, were predicted to be non-R5 [25], [28].

### Statistical analyses

Descriptive results are expressed as median values, interquartile ranges, and standard deviations. Pearson correlation coefficient was used to determine the strength of association between categorical variables. All differences with a *P* value of <0.05 were considered statistically significant. The kappa coefficient, which assesses a chance-adjusted measure of the agreement between any number of categories, was calculated using ComKappa2 v.2.0.4 [46] to quantify the concordance among the different the HIV-1 tropism determinations and the patient's virologic response at week 12. Values of kappa can range from −1.0 to 1.0, with −1.0 indicating perfect disagreement below chance, 0.0 indicating agreement equal to chance, and 1.0 indicating perfect agreement above chance. A rule of thumb is that kappa values <0.40 indicate poor agreement, ≥.40 <0.75 indicate good agreement, and ≥0.75 <1.0 indicate excellent agreement. All statistical analyses were performed using GraphPad Prism v.5.01 (GraphPad Software, La Jolla, CA) unless otherwise specified.

## Results

### Clinical samples

Twelve representative specimens were selected from 167 patients analyzed in a study comparing phenotypic and genotypic HIV-1 coreceptor tropism assays [13] based on the level of concordance among the different tests. Eleven samples corresponded to patients participating in the maraviroc EAP, with three failing to enter the study following the detection of non-R5 (D/M) variants at baseline and five out of the remaining eight patients responding to the maraviroc-based regimen at week 12 (Table 1). Population sequencing was performed not only to infer HIV-1 tropism but also to verify sample identity, showing a 99.9% homology with the V3 nucleotide sequences published for these specimens [21] (data not shown).

**Table 1:** Clinical and virological parameters of HIV-infected individuals.

Sample	Study^a^	CD4^+^ T-cell count (cells/ml) at		Plasma Viral Load (copies/ml) at		Virologic response at	HIV-1 tropism
		Baseline	Week 12	Baseline	Week 12	week 12^b^	at baseline^c^
10–65	EAP	166	18	1,500,000	437,000	No	R5
10–69	EAP	230	388	9,476	49	Yes	R5
10–73	EAP	268	225	15,778	39,995	No	R5
10–75	EAP	211	n.d.	27,200	n.d.	n.a.	R5
10–80	EAP	n.d.	n.d.	n.d.	n.d.	End of Study	D/M
10–91	EAP	28	239	168,000	39	Yes	R5
10–105	Allegro	n.d.	n.d.	n.d.	n.d.	n.a.	n.d.
10–133	EAP	30	250	56,445	96	Yes	R5
10–137	EAP	353	420	n.d.	49	Yes	R5
10–172	EAP	n.d.	n.d.	n.d.	n.d.	End of Study	D/M
10–176	EAP	n.d.	n.d.	n.d.	n.d.	End of Study	D/M
10–180	EAP	204	338	8,800	180	Yes	R5

a EAP, Expanded Access Program for the use of maraviroc at multiple centers; Allegro, multicenter study to assess the prevalence of R5 HIV-1 variants in Spain [39].

b Virologic response at week 12 following a maraviroc-based antiretroviral regimen, defined as a decrease in plasma viral load below 400 copies/ml. End of study, HIV-infected individuals did not enter the study following the detection of non-R5 viruses at baseline (see below); n.a., not available.

C HIV-1 coreceptor tropism was determined at baseline using a phenotypic test, i.e., the original Trofile™ assay (Monogram Biosciences) [11]. R5, CCR5-tropic virus; D/M, dual mixed, that is a combination of CCR5- and CXCR4-tropic viruses; n.d., not determined.

### Data processing and NGS platform error rates

**Figure 2:**
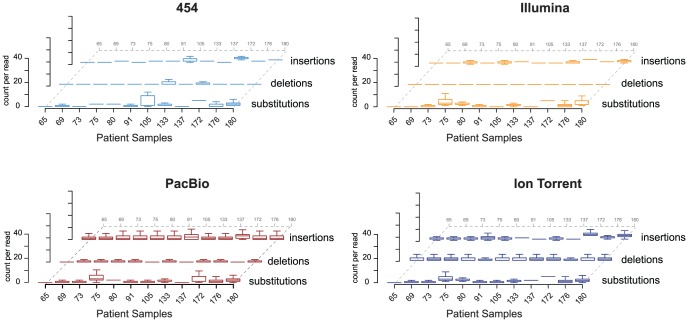
Comparison of data processing across NGS platforms. Number of sequencing errors, substitutions, deletions, and insertions (per read) for the NGS platforms: 454™, Illumina®, PacBio®, and Ion Torrent™. The mean and interquartile range (IQR) are indicated for each sample. Whiskers indicate 1.5 times the IQR as is the default value in the R-statistical package [75].

For each platform, the number of reads containing successfully mapped and complete V3 sequences, as well as those that were subsequently translated, were calculated. All insertions, deletions, and substitutions relative to the sample-specific reference sequences were tabulated during read mapping, and prior to any filtering based on correctly translating entire V3 sequences (Table 2). In general, all platforms showed low substitution (1.8, 1.7, 1.3, and 1.7 mean #/read), deletion (0.2, 0.01, 0.4, and 0.5 mean #/read), and insertion (2.1, 1.7, 1.9, and 2.4 mean #/read) rates across all samples for 454™, Illumina®, PacBio®, and Ion Torrent™, respectively (Fig. 2 and Table 2). As expected, there was interpatient variability; for example, sample 10–172 showed slightly higher substitution (5.1, 5.1, 2.5, and 5.1 mean #/read) and insertion (4.5, 4.0, 2.9, and 5.5 mean #/read) rates across all platforms compared with the other samples. Nevertheless, the overall low rates of indels and substitutions resulted in a high number of successfully translated V3 sequences from the original V3 spanning reads, suggesting that each one of the NGS platforms could be used for genotyping of complex HIV-1 populations.

**Table 2:** Cross platform rate variation.

Platform	Sample	No. V3 sequences before filtering	No. V3 sequences after filtering	Mean read length	Mean No. of substitutions per read	Mean No. of deletions per read	Mean No. of insertions per read
**454**	65	1533	1462	205.73	0.135	0.025	1.068
	69	1338	1013	204.79	0.311	0.204	1.099
	73	1388	1065	207.28	0.136	0.201	2.054
	75	2390	2058	201.12	2.182	0.086	1.216
	80	1231	1114	204.87	2.095	0.016	2.098
	91	918	851	213.20	0.349	0.015	2.075
	105	1363	1291	206.57	6.503	1.454	3.173
	133	1441	1131	201.42	1.574	0.191	1.929
	137	1698	1105	191.62	0.071	0.340	1.050
	172	1735	1640	203.19	5.070	0.014	4.451
	176	1451	1354	202.32	1.209	0.020	2.072
	180	1556	1158	201.83	2.287	0.096	3.277
	**Mean**	1504	1270	203.66	1.827	0.222	2.130
	**SD** a	352	326	5.05	2.056	0.401	1.046
	**Min**	918	851	191.62	0.071	0.014	1.050
	**Max**	2390	2058	213.20	6.503	1.454	4.451
**Illumina**	65	27194	26644	139.31	0.268	0.008	1.041
	69	24630	24097	137.55	0.381	0.009	1.034
	73	12947	12528	134.26	0.567	0.013	1.315
	75	9676	9454	136.91	3.637	0.013	1.058
	80	33184	32629	140.78	2.340	0.007	1.642
	91	18140	17586	140.58	0.560	0.011	1.983
	105	20516	19967	134.84	0.377	0.014	1.072
	133	8875	8538	137.03	1.562	0.020	1.314
	137	12803	12462	140.95	0.243	0.013	1.330
	172	17313	17088	136.53	5.120	0.012	4.027
	176	23114	22718	133.18	1.337	0.009	1.885
	180	5798	5638	137.63	3.973	0.017	2.255
	**Mean**	17849	17446	137.46	1.697	0.013	1.663
	**SD** a	8234	8120	2.57	1.690	0.004	0.849
	**Min**	5798	5638	133.18	0.243	0.008	1.034
	**Max**	33184	32629	140.95	5.121	0.020	4.027
**PacBio**	65	21190	8646	336.23	0.160	0.357	1.604
	69	20342	7279	335.77	0.478	0.472	1.684
	73	15428	5571	337.97	0.366	0.378	1.764
	75	18721	6776	336.80	3.603	0.413	1.814
	80	19812	6657	337.52	2.109	0.381	2.030
	91	16942	6350	338.11	0.322	0.251	1.816
	105	20582	6910	336.25	1.2919	0.585	2.516
	133	16243	6367	335.72	1.478	0.335	1.665
	137	21411	8162	340.27	0.077	0.433	1.571
	172	16533	5567	334.97	2.532	0.392	2.938
	176	8609	2490	334.12	1.073	0.280	2.083
	180	19070	5878	333.78	2.392	0.619	1.751
	**Mean**	17907	6388	336.46	1.324	0.409	1.937
	**SD** a	3567	1547	1.83	1.130	0.109	0.409
	**Min**	8609	2490	333.78	0.077	0.251	1.571
	**Max**	21411	8646	340.28	3.603	0.620	2.938
**Ion Torrent**	65	2662	1637	137.09	0.345	0.629	2.182
	69	2165	1187	136.67	0.520	0.672	1.799
	73	1720	1217	134.59	0.466	0.380	1.629
	75	1418	855	127.31	3.496	0.488	2.298
	80	1883	790	133.23	2.251	1.072	2.101
	91	1271	792	134.42	0.432	0.399	2.652
	105	2090	1703	139.11	0.337	0.211	1.480
	133	826	574	128.11	1.473	0.319	1.750
	137	517	436	103.31	2.017	0.197	1.251
	172	51	18	105.69	5.117	0.176	5.529
	176	2981	1874	141.98	1.268	0.283	2.842
	180	3404	691	140.89	2.197	1.126	3.971
	**Ave**	1749	981	130.20	1.660	0.496	2.457
	**SD** a	1000	557	12.80	1.476	0.323	1.212
	**Min**	51	18	103.31	0.337	0.176	1.251
	**Max**	3404	1874	141.98	5.117	1.126	5.529

a SD, standard deviation; Min, minimum value; Max, maximum value.

### HIV-1 population diversity

**Figure 3:**
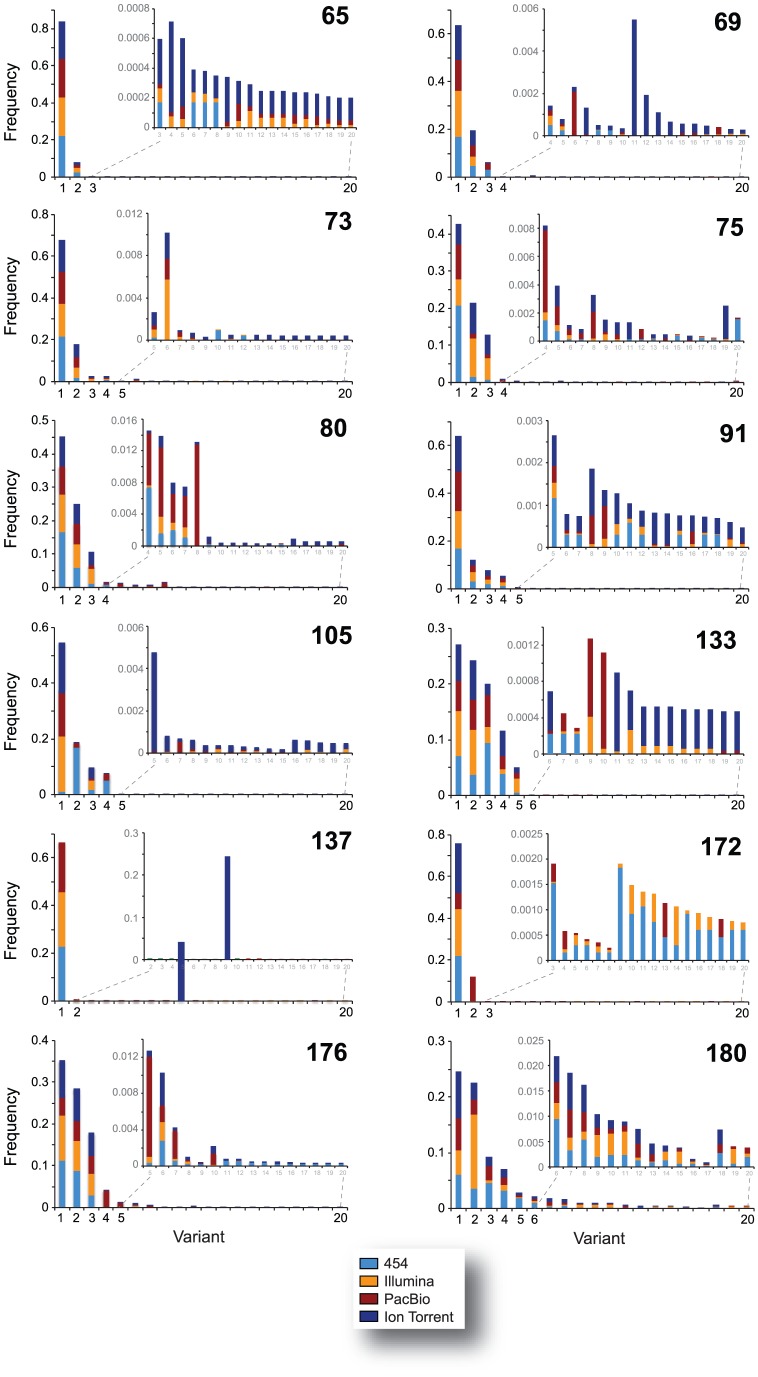
Comparison of the frequency of variants across NGS platforms. The heights of the bars represent the combined frequency of V3 variants detected by the NGS platforms 454™, Illumina®, PacBio®, and Ion Torrent™ prior to filtering. The colors within each bar denote the proportional contribution made by each platform after normalization based on coverage. Insets show low frequency variants up to a maximum of 20 unique sequences.

**Figure 4:**
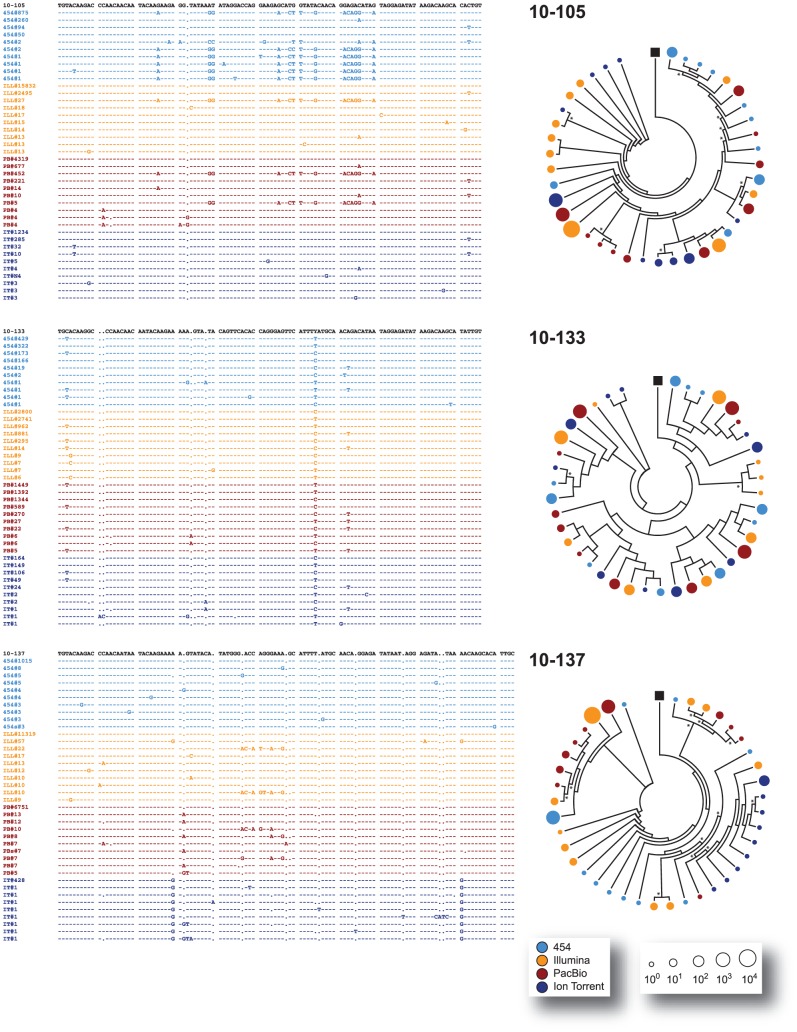
Comparison of the clustering of variants across platforms. The ten most common nucleotide V3 sequences from samples 10–65, 10–69, and 10–73 -obtained with each of the four NGS platforms (454™, Illumina®, PacBio®, and Ion Torrent™)- were aligned against the respective population (sanger) sequence from the respective patient using Clustal X 2.0 [76]. For each patient, every unique variant is identified by the NGS platform used and the number of sequences (frequency) obtained, e.g., 454#1290. For each position only those nucleotides that differ from the population sequence are depicted. Dashes indicated the same nucleotide as the population sequence while gaps introduced to maintain the alignment are indicated by dots. Relative clustering of the data from the NGS platforms was inferred by neighbor-joining, phylogenetic analyses determined using MEGA 5.05 [77] and displayed in a circle with topology only to facilitate their interpretation. Bootstrap resampling (1,000 data sets) of the multiple alignments tested the statistical robustness of the trees, with percentage values above 60% indicated by an asterisk. The size of the circles in the phylogenetic trees correlates with the frequency of the unique sequence determined by each NGS platform (color) in the logarithmic scale. The black box denotes the population (sanger) sequence for each sample.

**Figure 5:**
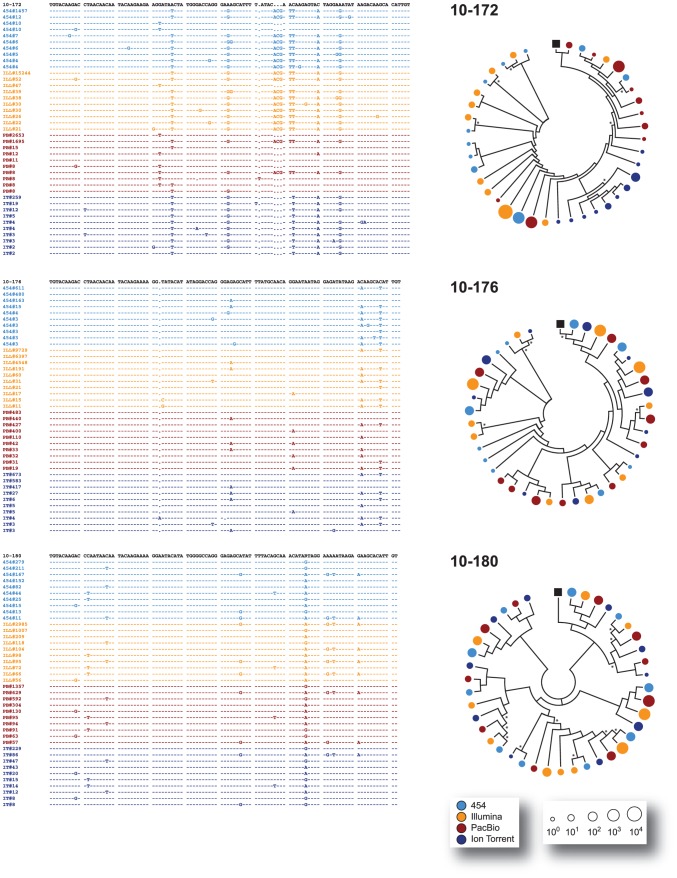
Comparison of the clustering of variants across platforms. The ten most common nucleotide V3 sequences from samples 10–75, 10–80, and 10–91 -obtained with each of the four NGS platforms (454™, Illumina®, PacBio®, and Ion Torrent™)- were aligned against the respective population (sanger) sequence and analyzed as described in the Figure 4 legend.

**Figure 6:**
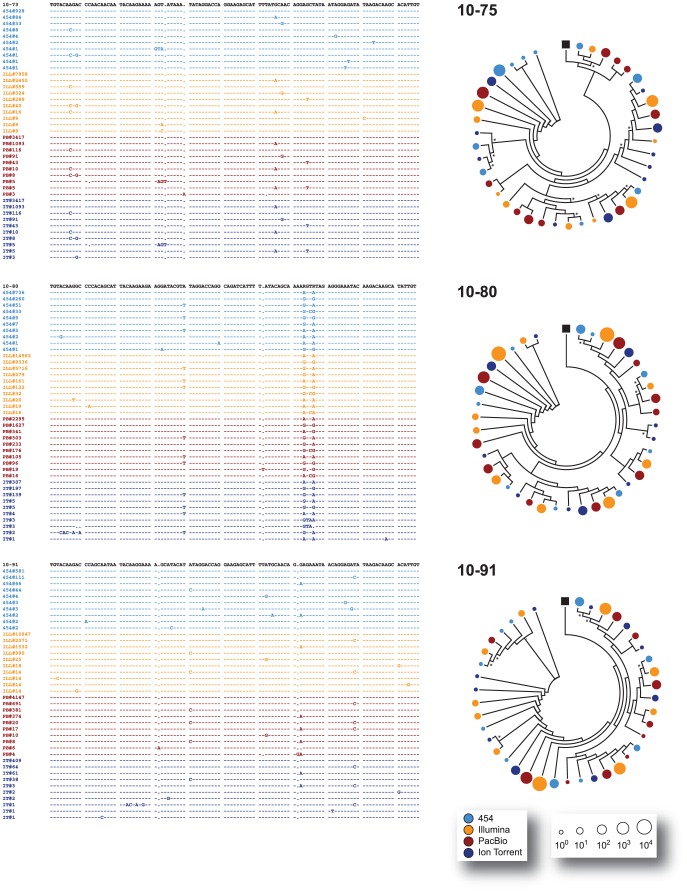
Comparison of the clustering of variants across platforms. The ten most common nucleotide V3 sequences from samples 10–105, 10–133, and 10–137 -obtained with each of the four NGS platforms (454™, Illumina®, PacBio®, and Ion Torrent™)- were aligned against the respective population (sanger) sequence and analyzed as described in the Figure 4 legend.

**Figure 7:**
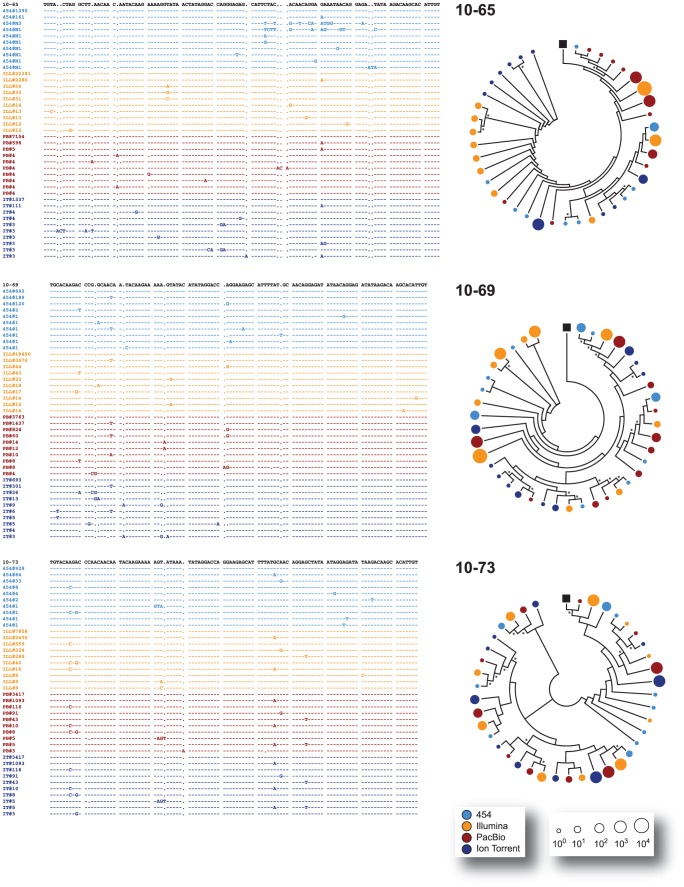
Comparison of the clustering of variants across platforms. The ten most common nucleotide V3 sequences from samples 10–172, 10–176, and 10–180 -obtained with each of the four NGS platforms (454™, Illumina®, PacBio®, and Ion Torrent™)- were aligned against the respective population (sanger) sequence and analyzed as described in the Figure 4 legend.

All major virus variants were detected by each platform with similar frequency, with the exception of sample 10–137 where Ion Torrent detected a different dominant variant than the other three platforms or in sample 10–172 where an insertion of three nucleotides was observed in most variants sequenced with 454, Illumina, and PacBio but not with Ion Torrent (Figs. 3, 4, and 5). All the amplicons, either the 337-nt fragments encompassing the V3 region or the 2,302 nt fragments covering most of the *env* gene, were obtained from the same external PCR products; however, the amplicons sequenced by Ion Torrent were generated seven months later than the products analyzed by the other NGS platforms. Thus, it is possible that in some cases (such as with sample 10–137) a different majority variant within the quasispecies population may have been selected during PCR amplification. Moreover, in some cases and at lower frequencies, unique variants were platform-dependent, probably related to platform-dependent error rates and/or stochastic PCR errors (Fig. 3). Nevertheless, and in general, all platforms were able to identify the same major variants within the population and similar proportion of low frequency variants (i.e., at a frequency <0.5%) (Fig. 3, inserts). For example, mixtures of at least two predominant populations were accurately identified by all four NGS platforms in samples 10–80, 10–133, and 10–180 (Figs. 3, 4, 5, and 6). Phylogenetic trees constructed by combining V3 sequences obtained with each platform confirmed these findings (Figs. 4, 5, 6, and 7).

### HIV-1 tropism determination by deep sequencing relative to Trofile™ and population-based sequencing

**Figure 8:**
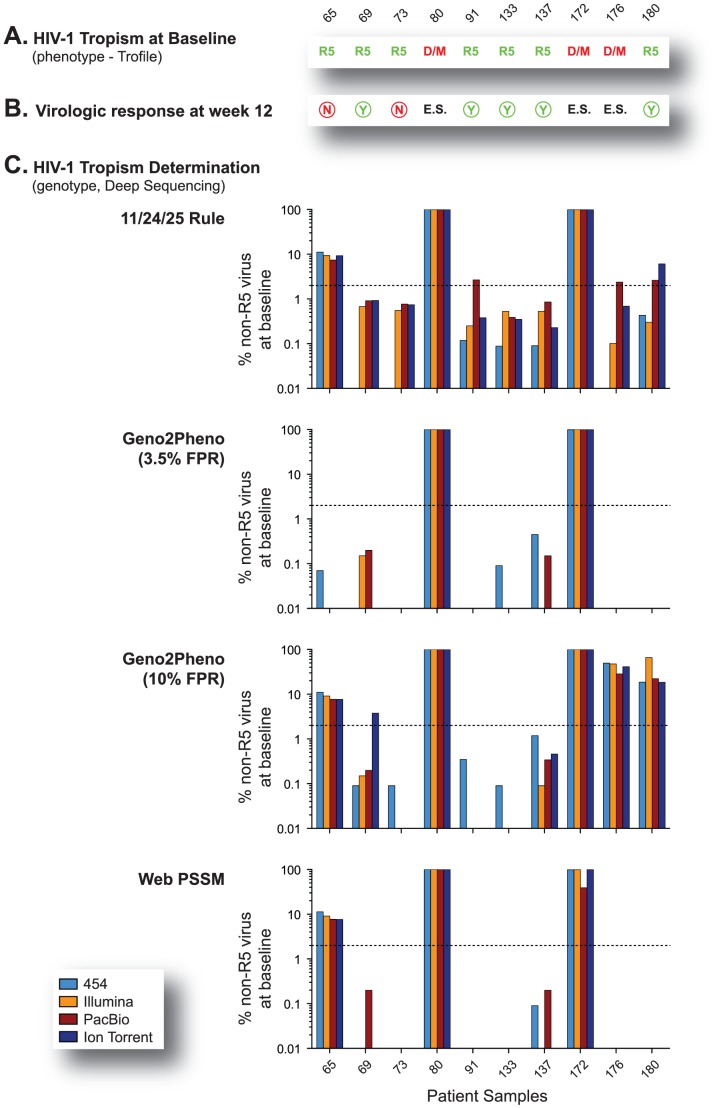
HIV-1 coreceptor tropism determination using deep sequencing. (A) HIV-1 tropism determined at baseline using Trofile™ (Monogram Biosciences) [11]; R5, CCR5-tropic virus; D/M, dual mixed. (B) Virologic response at week 12 of a maraviroc-based antiretroviral regimen. Y or N corresponds to plasma viral load below or not 400 copies/ml at week 12, respectively. E.S., end of study (patient did no enter the study following the detection of non-R5 variants at baseline using Trofile™). (C) Quantification of non-R5 variants detected by deep sequencing as predicted using four HIV-1 tropism algorithms, i.e., 11/24/25 rule [24], Geno2Pheno 3.5% FPR [25], [28], [42], Geno2Pheno 10% FPR, and Web PSSM using the subtype B x4r5 matrix [43]. Dotted line represents the ≥2% suggested cutoff for the minimal amount of non-R5 sequences to be present in the viral population in order to classify a given virus as non-R5 [25], [28].

The main goal of this study was to evaluate the ability of the four NGS platforms to determine HIV-1 coreceptor tropism. For that, the ten samples with known virologic response at week 12 (Table 1) were selected to compare the results from phenotypic and genotypic (population and deep sequencing) HIV-1 tropism assays, the latest using four different algorithms to predict HIV-1 coreceptor usage. Minority non-R5 variants were detected at comparable levels with a few exceptions mainly linked to the algorithm used to infer HIV-1 tropism rather than the NGS platform. For example, PacBio® (samples 10–91, 10–176, and 10–180; 11/24/125 rule) and Ion Torrent™ (samples 10–180 and 10–69; 11/24/25 rule and Geno2Pheno, respectively) detected a higher frequency of non-R5 variants than the other NGS systems (Fig. 8).

Interestingly, only the two samples carrying almost exclusively X4 viruses (10–80 and 10–172) were classified as non-R5 by all four algorithms (i.e., 11/24/25, Geno2Pheno 3.5% FPR, Geno2Pheno 10% FPR, and PSSM) based on a frequency of non-R5 variants ≥2% within the viral population. The most stringent Geno2Pheno 3.5% FPR failed to call one sample determined as D/M by Trofile (10–176) and specimens from two patients with virologic failure at week 12 (10–65 and 10–73). Not surprisingly, Geno2Pheno 10% FPR was able to call two of these samples as non-R5 (10–65 and 10–176) but also classified a patient with virologic success (10–180) as carrying non-R5 variants (Fig. 8). Finally, despite the limited sample number, prediction of HIV-1 coreceptor usage by all four NGS platforms showed similar concordance with the virologic response at week 12, ranging from 75% to 80% (kappa coefficients of 0.5 to 0.6, *P*<0.001) depending on the algorithm used, compared to Trofile (80%, kappa coefficient of 0.6) and population sequencing (mean 70%, kappa coefficient 0.4).

## Discussion

The use of CCR5 antagonists to block HIV-1 replication has accelerated the development of HIV-1 coreceptor tropism assays [7], [8], [9] and stressed the need for novel, sensitive, and more affordable tests to increase treatment with this drug class. Although Trofile is the most commonly used phenotypic assay for HIV-1 coreceptor tropism, less sensitive genotypic tests based on HIV-1 population (Sanger) sequencing are frequently used in Europe, leading to the rapid adoption of deep sequencing technologies in genotypic HIV-1 coreceptor tropism protocols [22], [23], [24], [25], [27], [28], [34], [35], [36], [47]. Based on the need for these NGS-based genotypic assays, we have compared the ability of four NGS platforms (454™, Illumina®, PacBio®, and Ion Torrent™) to detect minority variants, and to infer the presence of non-R5 viruses within the HIV-1 population.

Next generation sequencing has been used in a multitude of biological fields, from the sequencing of whole genomes of animals, plants, and microbes, to targeted studies on polymorphisms related to various genetic disorders and cancer, most of them based on Illumina® [48], [49], [50], [51] and 454™ [52], [53], [54] platforms and more recently using PacBio® [55], [56], [57] and Ion Torrent™ [58], [59], [60] systems. To date all published HIV-related studies have used the 454™ platform, due in part to being one of the first NGS systems to provide longer read lengths [53], [61]. As expected each deep sequencing platform differs in terms of the chemistry, read length, yield, error rate, turn-around time, and overall cost [62], [63]. Here, we sequenced the HIV-1 V3 region from the same RNA aliquots obtained from 12 patients and showed that all four NGS platforms had similar substitution and insertion rates (ranging from 1.3 to 1.8 and 1.7 to 2.4 mean #/read, respectively), while Illumina® had the fewest deletions per read (0.01 versus a range of 0.2–0.5 mean #/read for the other three platforms). This is consistent with the reduced number of indels reported for Illumina® when compared with 454™ during the genome sequencing of *Gallus gallus* [64] or influenza virus [40] and the sequencing of a strain of *Escherichia coli* using 454™ and Ion Torrent™ [65].

We observed differences in the number of V3 sequences and mean read length among the NGS platforms, which were due to both the size of the PCR product selected for sequencing and intrinsic characteristics of the sequencing method. Short amplicons (337 nt) containing the V3 region were sequenced using 454™ and PacBio®; however, library preparation and shotgun sequencing was performed on the entire PCR-amplified *env* gene (2,302 nt) using Illumina® and Ion Torrent™. In addition, the 454™ sequencing was performed in-house using barcoded sequencing primers while for Illumina®, PacBio®, and Ion Torrent™ the amplicons were sent for sequencing at the respective company. Despite these differences, all NGS platforms were able to detect the same higher frequency variants but showed slightly variations in the detection of low frequency variants (<0.5%), which had limited implications for HIV-1 tropism. It is important to note that based on HIV-1 clonal sequencing the error rate for in-house 454™ sequencing assays has been calculated to be between 0.1% and 0.5% [23], [34], [66], [67]; therefore, we only used variants present at ≥1% of the viral population for diversity and HIV-1 tropism analyses.

Multiple studies have compared the efficacy of phenotypic and genotypic HIV-1 tropism assays to detect non-R5 variants [68], [69], [70], [71], [72]. In general, population-based sequencing tests are less sensitive and less specific than phenotypic assays [7], [73], although a few studies have shown significant concordance and predictive values [28], [72], [74]. More sensitive deep sequencing methods for HIV-1 coreceptor tropism assays resulted in the detection of minor variants, which correlated well with both phenotypic assays [13], [25], [28], [34] and virological response to maraviroc [25], [28]. Here we have shown that all four NGS platforms provide equal and sensitive detection of minority non-R5 viruses in 12 patients, with minor differences depending on the bioinformatic algorithm used to infer HIV-1 tropism. However, it is important to stress that maraviroc was combined with at least two other antiretroviral drugs and increased viral loads could be due to several factors including poor or selective drug adherence and resistance to other drugs while maintaining partial maraviroc suppression. Nevertheless, all four NGS platforms showed similar concordance with virologic response at week 12 (ranging from 75% to 80% depending on the algorithm used), compared to Trofile (80%) and population sequencing (70%). Despite the limited number of samples, these results are comparable to previous studies where deep sequencing had a good concordance with phenotypic HIV1- tropism tests (82% to 87%) [25], [28], [34] and matched Trofile™ in predicting the success of maraviroc-based antiretroviral regimens [28].

In conclusion, this is the first study comparing the ability of the four current leaders in deep sequencing (454™, Illumina®, PacBio®, and Ion Torrent™) to detect minority variants, and to infer the presence of non-R5 viruses, within the HIV-1 population. Despite minor differences in error rates and profiles (types of errors), all four NGS platforms successfully detected the same unique viral variants present at high frequencies, which are the sequences relevant for the clinical determination of HIV-1 coreceptor tropism. Further studies with larger number of patients and the latest chemistry and software for each NGS system will be needed to corroborate our findings; however, despite intrinsic parameters to each NGS platform (e.g., read length, error rates, cost per run, and turn-around time) we suspect that any of the current NGS platforms will be effective in a genotypic test to predict HIV-1 coreceptor usage.
